# Clinical Utility of Anti-Gliadin IgG Antibody (AGA IgG) and Characterization of Patients with Suspected Non-Celiac Gluten Sensitivity: Prospective, Observational Study in Japan

**DOI:** 10.3390/nu18101607

**Published:** 2026-05-18

**Authors:** Mikuni Motoyama, Hisashi Yamada, Chiho Yoshimura, Hisato Matsunaga

**Affiliations:** Department of Neuropsychiatry, Hyogo Medical University, 1-1, Mukogawa, Nishinomiya 663-8501, Japan; hisa0820@hyo-med.ac.jp (H.Y.); y-chiho@hyo-med.ac.jp (C.Y.); hisa1311@hyo-med.ac.jp (H.M.)

**Keywords:** non-celiac gluten sensitivity (NCGS), gluten sensitivity, gluten intolerance, anti-gliadin IgG antibody (AGA IgG), biomarker, gluten-related disorders

## Abstract

Background/Objectives: Non-celiac gluten sensitivity (NCGS) is a syndrome characterized by intestinal and extraintestinal symptoms triggered by gluten ingestion. Although anti-gliadin IgG antibody (AGA IgG) has been proposed as a potential biomarker for NCGS, its sensitivity and specificity in real-world clinical settings remain unclear. This study aimed to evaluate the clinical utility of AGA IgG in NCGS and to characterize its clinical features, including psychological distress and physical quality of life (QOL), in patients with clinically suspected NCGS attending a specialized outpatient unit in Japan, where patients reported symptoms related to the ingestion of gluten-containing grains (primarily wheat). Methods: We evaluated plasma AGA IgG levels in 45 patients with suspected NCGS based on clinical presentation and in 83 age- and sex-matched healthy controls. Plasma AGA IgG was measured using ELISA. Clinical symptoms and QOL were assessed using validated scales, including the 36-Item Short Form Health Survey (SF-36), Patient Health Questionnaire (PHQ-9 and PHQ-15), Generalized Anxiety Disorder-7 (GAD-7), and the Japanese version of the Irritable Bowel Syndrome Quality of Life measure (IBS-QOL-J). Results: The AGA IgG positivity rate was significantly higher in the suspected NCGS group (33.3%) than in the control group (13.3%; *p* < 0.01). Using clinical suspicion as the reference, the sensitivity and specificity of AGA IgG were 33.3% and 86.7%, respectively. Patients with suspected NCGS exhibited significantly lower physical and mental QOL and higher scores for depressive, anxiety, and somatic symptoms compared to controls. No significant clinical differences were found between AGA IgG-positive and IgG-negative individuals within the suspected NCGS group. Conclusions: AGA IgG demonstrated a specificity of 86.7% and a sensitivity of 33.3% for suspected NCGS, indicating its limited utility as a standalone biomarker. These findings suggest that suspected NCGS involves significant somatic and psychological burdens regardless of serological status. Future studies should explore whether a multi-marker panel could improve the identification of “True NCGS” in diverse clinical populations.

## 1. Introduction

Gluten is a protein network formed when wheat, barley, rye, and spelt are hydrated and mechanically agitated. In wheat, it is formed by the combination of glutenin and gliadin, whereas the corresponding prolamins in barley and rye are hordein and secalin, respectively [1]. Beyond its physical properties, gluten plays a critical role in food production by influencing the sensory, technological, and nutritional quality of products. These characteristics represent the primary obstacles to excluding gluten from food manufacturing and the daily diet, as replicating the structural and palatable properties of gluten remains a significant challenge [2].

Non-celiac gluten sensitivity (NCGS) is a syndrome characterized by intestinal and extraintestinal symptoms triggered by the ingestion of gluten [3]. However, it has been increasingly recognized that other components of wheat, such as amylase-trypsin inhibitors (ATIs) and fermentable oligo-, di-, and monosaccharide and polyol foods (FODMAPs), may also trigger similar clinical manifestations. Consequently, the broader term non-celiac wheat sensitivity (NCWS) is often used to encompass these diverse triggers [4]. While this study primarily employs the widely recognized term NCGS, we acknowledge the inherent heterogeneity of the condition and the potential involvement of non-gluten components. The common intestinal symptoms of NCGS include diarrhea, bloating, abdominal pain and nausea, while extraintestinal symptoms include headache, anxiety, depression, brain fog, and fatigue [3]. NCGS is one of the gluten-related disorders, distinct from autoimmune diseases such as celiac disease or gluten ataxia, and allergic diseases such as food allergy or wheat-dependent exercise-induced anaphylaxis [5]. It is also referred to as gluten intolerance or gluten sensitivity. There may be an overlap between NCGS and food protein-induced enterocolitis syndrome in adults caused by wheat, as well as irritable bowel syndrome (IBS), due to partial similarities in clinical presentation. Some patients previously classified as having IBS and improving on a gluten-free diet (GFD) may have had unrecognized NCGS or other wheat-related sensitivity [6,7]. On the other hand, NCGS is the subject of debate as to whether it even exists as an independent disease due to the absence of diagnostic criteria, validated biomarkers, and the high variability of symptoms among individuals, and the wide range of symptoms. In fact, a randomized, double-blind, placebo-controlled trial investigating the effects of anticipation of gluten intake and actual gluten consumption on symptoms in NCGS patients found that those who believed that gluten was present experienced more severe gastrointestinal symptoms, regardless of its actual presence. Specifically, 56% of participants reported symptoms even when consuming a placebo, suggesting a nocebo effect [8]. These findings underscore the importance of distinguishing biological responses to various wheat components from psychological factors.

NCGS was first reported in the 1980s [9], and while various studies have been conducted, the pathophysiology still remains unclear. Diagnostic criteria, including definitive biomarkers for NCGS, have not been determined yet. Previous studies have shown that anti-gliadin IgG antibodies (AGA IgG) are the most frequently positive antibodies in NCGS patients and are considered a potential biomarker. We selected AGA IgG as a primary parameter because it is thought to reflect a systemic immune response to gluten proteins that have crossed the intestinal epithelial barrier due to increased intestinal permeability. This process is considered a key hypothesized mechanism in the development of NCGS [10,11]. Unlike the highly specific autoantibodies used to diagnose celiac disease such as anti-tissue transglutaminase (tTG), AGA IgG may capture the more subtle immune activation characteristic of non-autoimmune gluten sensitivity. However, recent clinical perspectives have increasingly highlighted that traditional diagnostic tools, including AGA IgG, often fail to provide a definitive diagnosis due to their limited specificity and sensitivity [12]. Indeed, AGA IgG are positive in only 25–55% of NCGS patients [13,14,15] and its clinical utility has never been investigated in detail. The “Salerno Experts’ Criteria”, a diagnostic process for NCGS proposed by Salerno et al. [3], diagnoses NCGS by evaluating the changes in symptoms through repeated gluten challenge and gluten-free instances. However, the Salerno Experts’ Criteria is not easy and impractical to use for the diagnosis of NCGS in daily clinical practice for both clinicians and patients because it takes at least 9 weeks to make a determination, it is difficult to perform gluten challenge and gluten-free strictly, and gluten challenge is often difficult due to various symptoms. In addition, the assessment of symptoms is subjective, and objective measures have not been utilized.

Moreover, because diagnostic criteria and reliable biomarkers for NCGS have yet to be established, many individuals initiate a GFD based on self-diagnosis and without medical supervision. This trend raises concerns from a nutritional perspective. Long-term, self-prescribed adherence to a GFD has been associated with inadequate intake of dietary fiber, micronutrients, and essential minerals, thereby potentially increasing the risk of nutritional deficiencies [16]. Furthermore, reliance on commercially available gluten-free products may lead to excessive caloric intake and weight gain, as these products often possess a higher glycemic index and increased lipid content to compensate for the functional absence of gluten [17]. These factors combined potentially increase the risk of both nutritional deficiencies and metabolic imbalances.

Based on epidemiological data from multiple regions, including North America, South America, and Europe, the prevalence of NCGS is reported to range from 0.5% to 15% [18,19,20,21]. Due to the lack of sensitive and reproducible biomarkers and clinical diagnostic criteria, each previous study had used different criteria of NCGS or evaluated the prevalence of NCGS based only on self-report. Thus, the exact epidemiology of NCGS is uncertain.

Hyogo Medical University Hospital, situated in Nishinomiya, Japan, started a specialized outpatient unit for suspected NCGS in 2020. The hospital is positioned in the Hanshin region, which is a major metropolitan area. We have examined patients suspected of NCGS to clarify and establish biomarkers and diagnostic criteria for NCGS. Given that many patients initiate a GFD without medical guidance, there is an urgent clinical need for objective indicators to prevent inappropriate nutritional restrictions and metabolic risks. We hypothesized that plasma AGA IgG levels would correlate with the clinical symptoms of patients suspected of NCGS, and that this biomarker could serve as a practical tool for identifying individuals who are most likely to benefit from a GFD. Therefore, the aim of this study was to assess patients who attended our outpatient unit for suspected NCGS objectively, using gluten-related antibodies, and to investigate the association between AGA IgG and their clinical symptoms. In addition, by examining the usefulness, sensitivity and specificity of AGA IgG, we sought to improve the precision of NCGS diagnosis and prevent unnecessary, nutritionally imbalanced dietary restrictions.

## 2. Method

### 2.1. Study Design and Participants

This study was a prospective, observational study. Reporting of this exploratory clinical investigation was informed by clinical research principles to ensure transparency and comprehensive data presentation. We enrolled patients with gluten intolerance residing in Japan who attended the specialized NCGS outpatient unit at Hyogo Medical University Hospital (Nishinomiya, Japan) between January 2020 and April 2023. The participant recruitment followed a specific clinical pathway: all patients first underwent an initial screening for gluten-related symptoms and a thorough clinical interview by physicians specializing in gluten-related disorders to confirm the onset or exacerbation of symptoms following gluten ingestion. The non-celiac patients who experienced intestinal or extraintestinal symptoms after gluten ingestion and reported symptom improvement on a GFD were categorized as having clinically suspected NCGS. Exclusion criteria included: (1) diagnosis of celiac disease, (2) wheat allergy, (3) severe hepatic disease, (4) use of immunosuppressants, (5) presence of eating disorders or unbalanced dietary habits unrelated to gluten, and (6) strict adherence to a GFD prior to enrollment that resulted in AGA IgG levels below the detection limit. Celiac disease was excluded based on clinical evaluation and serological testing. Specifically, individuals with positive results for tTG IgA antibodies were excluded. Although total IgA levels and HLA-DQ2/DQ8 status were not routinely assessed, this screening approach was considered clinically appropriate given the extremely low prevalence of celiac disease in the Japanese population [22]. For the control group, healthy volunteers were recruited through public advertisements within the university. We enrolled age- and sex-matched subjects without any history of psychiatric disorders. The absence of psychiatric disorders was confirmed using with the questionnaire of the Structured Clinical Interview for DSM, 5th edition, text revision (DSM-5-TR). The eligibility criteria for the control group also included: (1) no history of gluten related disorders, (2) no gastrointestinal or systemic symptoms related to wheat ingestion, and (3) no chronic inflammatory or autoimmune diseases. Individuals following a GFD were excluded. Furthermore, all participants underwent clinical evaluation by experienced physicians to rule out symptoms of malabsorption or history suggestive of IgA deficiency. Wheat allergy was ruled out by clinical evaluation. Specifically, individuals with positive wheat-specific IgE results (measured by fluorescence enzyme immunoassay) were excluded. Individuals with a history of immediate hypersensitivity, such as urticaria, angioedema, and respiratory distress, or wheat-dependent exercise-induced anaphylaxis were excluded. We confirmed that all participants did not have abnormal eating habits or an unbalanced diet, because a GFD may decrease AGA IgG concentration [6]. All subjects voluntarily provided written informed consent to participate in this study. Following consent, blood samples were collected for AGA IgG measurement, and clinical symptom questionnaires were completed on the same day. All the procedures of this study complied with the ethical standards of the relevant national and institutional committees on human experimentation and with the Helsinki Declaration of 1975, as revised in 2013. This study was approved by the ethics committee of Hyogo Medical University on 20 January 2020 (no. 3412). Detailed explanations of the study procedures were provided to each participant when informed consent was received. The study protocol was registered in UMIN-CTR (UMIN000038700).

### 2.2. Clinical Assessments

We objectively investigated both the suspected NCGS and control groups by measuring plasma AGA IgG levels in all participants and examined their demographic and clinical characteristics. At the initial assessment, demographic profiles, medical histories, and clinical features were obtained through face-to-face interviews. Specific parameters of all participants including age, sex, height, weight, presence or absence of food or drug allergies, and medical history were obtained. In addition, participants answered the self-administered questionnaires with Patient Health Questionnaire-9 (PHQ-9) to assess depressive symptoms [23], Patient Health Questionnaire-15 (PHQ-15) to assess somatic symptoms [23], Generalized Anxiety Disorder-7 (GAD-7) to assess anxiety [23], Japanese version of the irritable bowel syndrome quality of life (IBS-QOL-J) to assess quality of life related to gastrointestinal symptoms that are common among NCGS patients [24], and 36-Item Short Form Health Survey questionnaire (SF-36) to assess health-related quality of life (QOL) [25,26]. These instruments are standardized tools that have been validated and are widely used in clinical research in Japan.

### 2.3. Serological Analysis

AGA IgG levels were assayed in both patients with suspected NCGS and healthy controls. Blood samples were collected from all participants during their initial clinical evaluation. Plasma was then separated by centrifugation and stored for subsequent analysis. All plasma samples were frozen and stored at −30 °C until assayed. To ensure objective measurement and data collection, both the medical staff responsible for blood sampling and the laboratory personnel performing the ELISAs were blinded to the participants’ clinical status (NCGS group or healthy control group). Plasma was analyzed for AGA IgG antibody using RUO534G kit (Orgentec Diagnostika, Mainz, Germany), which is a commercially available enzyme-linked immunosorbent assay (ELISA) kit, in accordance with the manufacturer’s protocol. The limit of detection for AGA IgG was 0.5 U/mL, and a cutoff level of 12 U/mL was adopted as provided by the manufacturer. All samples were run in duplicate.

### 2.4. Statistical Analysis

The required sample size was determined using G*Power 3.1. Based on an effect size (w) of 0.3, an alpha level of 0.05, and a power (1 − β) of 0.80, a total sample size of at least 110 participants was required. Our final sample size of 128 (45 suspected NCGS and 83 controls) exceeded this requirement, ensuring adequate statistical power for the comparative analyses. We assessed the homoscedasticity of variance using Levene’s test. Because the Kolmogorov–Smirnov test indicated that the data were not normally distributed and the assumption of homoscedasticity was not met, the Mann–Whitney U test was employed to compare non-parametric variables. Pearson’s chi-square test was used to compare the proportions of categorical variables between the groups. For comparisons involving four groups, the Kruskal–Wallis test was used for continuous variables, followed by Dunn’s post hoc test with Bonferroni correction for pairwise comparisons. Regarding the comparison of proportions among the four groups, Haberman’s adjusted residual analysis was performed following a significant chi-square test. To account for multiple testing in this analysis, a Bonferroni-corrected significance threshold of ∣Z∣ > 2.58 was applied. Data were expressed as medians and interquartile ranges (IQRs) for continuous variables, and as frequencies and percentages for categorical variables. To evaluate the independent association between AGA IgG positivity and suspected NCGS, multivariable binary logistic regression analysis was performed. In this model, group classification (suspected NCGS vs. control) was set as the dependent variable. Potential confounders identified from previous literature and clinical relevance, including age, sex, BMI, allergy status, and psychiatric scores (GAD-7 and PHQ-9), were included as independent variables (covariates). The goodness-of-fit of the model was assessed, and odds ratios (ORs) with 95% confidence intervals (CIs) were calculated. All statistical analyses were performed using IBM SPSS, version 24.0 (IBM Corp., Armonk, NY, USA). All tests were two-tailed. To account for multiple comparisons, *p*-values were adjusted using the Bonferroni correction. Specifically, a significance threshold of *p* < 0.0083 was applied for primary clinical and QOL scales, and *p* < 0.0033 was used for individual somatic symptoms. For other demographic and baseline comparisons, a significance level of *p* < 0.05 was maintained.

## 3. Results

### 3.1. Participant Characteristics and Serological Status

**Figure 1:**
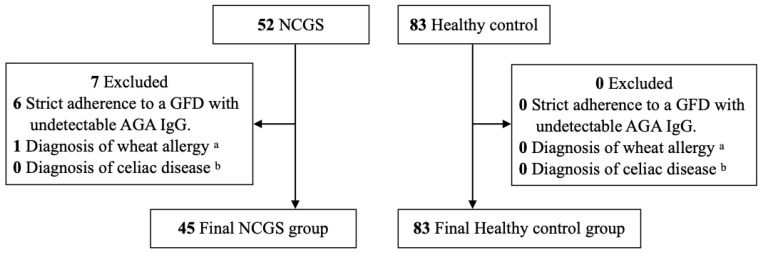
Flow of participants through the study. Out of 52 patients initially assessed, 7 were excluded (6 due to strict adherence to a GFD prior to enrollment that resulted in AGA IgG levels below the detection limit and 1 due to a wheat allergy). No patients met the diagnostic criteria for celiac disease. The final analysis included 45 patients with suspected NCGS and 83 age- and sex-matched healthy controls. AGA IgG, anti-gliadin IgG antibody; GFD, gluten-free diet; NCGS, Non-celiac gluten sensitivity. ^a^ Determined based on clinical symptoms and specific IgE measurement. ^b^ Confirmed by positivity for anti-tissue transglutaminase IgA antibodies.

The participant recruitment process is illustrated in Figure 1. Of the 52 patients initially assessed, 7 were excluded: 6 due to strict adherence to a GFD prior to enrollment, which resulted in AGA IgG levels below the detection limit, and 1 due to a diagnosis of wheat allergy. No patients met the diagnostic criteria for celiac disease. Consequently, the final study population included 45 patients with suspected NCGS and 83 age- and sex-matched healthy controls. In the suspected NCGS group, 15 patients (33.3%) were positive for AGA IgG, compared to 11 (13.3%) in the control group. The patients with suspected NCGS had a significantly higher rate of AGA IgG positivity than the control group (*p* = 7.0 × 10^−3^).

### 3.2. Clinical Utility of AGA IgG in Suspected NCGS

The clinical utility of AGA IgG as a biomarker for suspected NCGS was evaluated. Using clinical suspicion as the reference standard, the sensitivity was 33.3% (95% CI: 20.0–49.0%) and the specificity was 86.7% (95% CI: 77.5–93.2%). The positive predictive value (PPV) and negative predictive value (NPV) were 57.7% and 70.6%, respectively.

### 3.3. Participant Demographics and Baseline Characteristics

The demographic and clinical profiles of the study population are summarized in Table 1. There were no statistically significant differences between the suspected NCGS and control groups regarding median age, sex ratio, or body mass index. However, a significant difference was observed in the prevalence of allergic diseases (other than wheat allergy), which was markedly higher in the suspected NCGS group compared to that in healthy controls.

### 3.4. Impairment of QOL in NCGS

Patients with suspected NCGS exhibited a substantial reduction in health-related quality of life compared to the control group. Specifically, both physical and mental component summaries of the SF-36, as well as the total IBS-QOL-J score, were significantly lower in suspected NCGS patients. This impairment was consistent across all individual subscales of the SF-36 and IBS-QOL-J, highlighting the broad impact of gluten sensitivity on patient well-being.

### 3.5. Psychological and Somatic Symptom Burden

The burden of psychological and physical distress was significantly more pronounced in the suspected NCGS group. Scores on validated scales for depressive (PHQ-9), anxiety (GAD-7), and somatic symptoms (PHQ-15) were all significantly higher in patients with suspected NCGS than in healthy controls.

Detailed analysis of the PHQ-15 somatic symptoms is presented in Table 2. Individuals with suspected NCGS demonstrated a significantly increased likelihood of experiencing symptoms across the majority of categories. In addition to gastrointestinal manifestations such as nausea, gas, and altered bowel movements (including constipation and diarrhea), the suspected NCGS group frequently presented with highly significant extraintestinal symptoms, including fatigability, low energy, and sleep disturbances. Other symptoms, such as headaches and dizziness, were more prevalent in the NCGS group but did not reach statistical significance after the conservative multiple comparison adjustment.

### 3.6. Clinical Profiles and Predictive Factors for Suspected NCGS

To evaluate the clinical relevance of AGA IgG, we compared demographic characteristics and clinical symptoms across four subgroups stratified by suspected NCGS status and AGA IgG serostatus (Table 3). No significant differences were observed among the four groups regarding age, sex, or BMI. However, the prevalence of non-wheat allergies, diarrhea, and abdominal pain differed significantly across the groups. Haberman’s adjusted residual analysis revealed that both NCGS subgroups (AGA IgG-positive and -negative) showed a significantly higher prevalence of these symptoms compared to the control groups, regardless of their serostatus. There were significant differences across the four groups in all measures of psychiatric distress (PHQ-9, PHQ-15, and GAD-7) and QOL metrics (SF-36 component summaries and IBS-QOL-J). Post hoc pairwise comparisons demonstrated that both the AGA IgG-positive and AGA IgG-negative NCGS groups had significantly higher psychiatric distress scores and lower QOL scores compared to healthy controls. No significant differences were observed between the AGA IgG-positive and AGA IgG-negative NCGS patients in any of these clinical or psychological measures.

Subsequently, we identified independent factors associated with suspected NCGS using multivariable logistic regression analysis (Table 4). After adjusting for age, sex, BMI, allergy status, and psychiatric scores (GAD-7 and PHQ-9), AGA IgG positivity remained a significant independent predictor (*p* = 2.0 × 10^−2^, Odds Ratio = 5.7, 95% CI: 1.3–24.8). Allergy status (*p* = 4.0 × 10^−3^) and GAD-7 scores (*p* = 1.4 × 10^−2^) were also identified as independent factors. Logistic regression analysis further confirmed that AGA IgG positivity was not statistically associated with specific clinical or psychological features within the suspected NCGS cohort. Similarly, within the control group, no significant differences were found between AGA IgG-positive and IgG-negative individuals in any demographic or clinical parameters.

### 3.7. ROC Curve Analysis for Diagnostic Performance

To further evaluate the diagnostic potential of AGA IgG, a receiver operating characteristic (ROC) curve analysis was performed. The area under the curve (AUC) for distinguishing the suspected NCGS cohort from the control group was 0.62 (95% CI: 0.52–0.72, *p* = 2.4 × 10^−2^). Based on the Youden index, the optimal provisional cut-off value was determined to be 10.0 U/mL, yielding a sensitivity of 42.2% and a specificity of 84.1%. While this statistical threshold was slightly lower than the manufacturer-established cut-off (12.0 U/mL), it confirms the significant, albeit modest, discriminatory power of AGA IgG as a biological marker in this population.

## 4. Discussion

To date, several studies have investigated the positivity rate of AGA IgG in patients with NCGS, but data on its sensitivity and specificity remain limited. Our study provides these metrics (33.3% and 86.7%, respectively) within a clinical cohort. However, it is important to acknowledge that the present is characterized as a clinic-based exploratory investigation of patients with suspected NCGS. These values indicate that AGA IgG alone is insufficient for a definitive diagnosis of NCGS, as clinical symptoms did not differ significantly between AGA IgG-positive and -negative patients in the suspected group. However, we maintain that no single biomarker can currently serve as a stand-alone tool for NCGS due to its pathophysiological complexity. Instead, our findings highlight the necessity of combining multiple immunological and clinical markers to improve diagnostic accuracy in real-world practice. While some studies have suggested that AGA IgG lacks sufficient specificity for NCGS diagnosis because it can also be detected in patients with celiac disease, autoimmune disorders, and IBS [27], our study maintains its clinical relevance by focusing on a cohort where overt autoimmune and allergic conditions were clinically ruled out. In the context of a specialized outpatient unit in Japan, where wheat consumption is traditionally lower than in Western countries, identifying a specific immune response to gliadin remains a vital step in characterizing this unique patient population. We consider two possible explanations for these findings.

**Figure 2:**
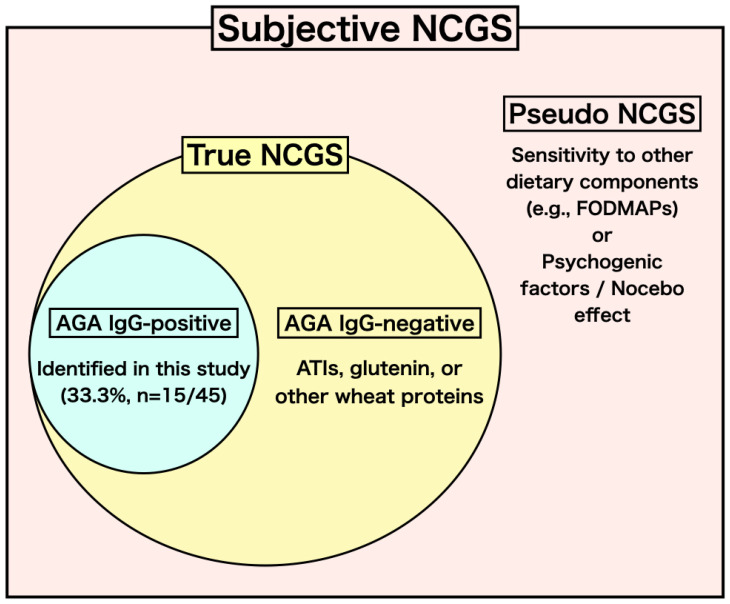
A hypothetical conceptual model for the subclassification of Non-Celiac Gluten Sensitivity (NCGS) based on immunological and clinical observations. “Subjective NCGS” encompasses all patients reporting symptoms triggered by wheat ingestion. “True NCGS” includes individuals with objective immune responses; our study identified 33.3% as AGA IgG-positive, while others may react to different wheat components such as ATIs or glutenin. “Pseudo NCGS” represents cases driven by the nocebo effect or sensitivities to non-wheat components (e.g., FODMAPs). This framework is proposed as a hypothesis-generating model for future clinical validation and does not imply that AGA IgG is the sole definitive marker for all cases. NCGS, non-celiac gluten sensitivity; AGA IgG, anti-gliadin IgG antibody; ATIs, amylase-trypsin inhibitors; FODMAPs, fermentable oligo-, di-, and monosaccharide and polyol foods.

First, NCGS is considered a heterogeneous condition. We propose a conceptual hypothesis regarding NCGS from the perspective of AGA IgG, as illustrated in the hypothetical model in Figure 2. Patients who experience the onset or worsening of symptoms after wheat consumption correspond to “Subjective NCGS” in Figure 2. In our study, 33.3% of patients tested positive for AGA IgG, representing a subgroup of patients with NCGS who specifically react to gliadin. While all participants in our specialized outpatient unit reported clinical symptoms specifically triggered by wheat ingestion, it is important to consider that the immune response in NCGS may extend beyond wheat-derived gliadin to other cereal prolamins, such as secalin in rye and hordein in barley. These prolamins share structural similarities with gliadin and are known to trigger similar clinical reactions in gluten-related disorders. Therefore, the 33.3% AGA IgG-positive rate identifies a specific immune-reactive phenotype within the broader spectrum of patients experiencing wheat-induced symptoms. Wheat contains not only gliadins, but also other proteins involved in immune responses, such as glutenin, ATIs, puroindolines, and purothionin. It is possible that some NCGS patients who are negative for AGA IgG react to these components (“AGA IgG-negative in True NCGS” in Figure 2). Indeed, ATIs have attracted attention recently. While ATIs have long been recognized as causal agents of occupational allergies such as baker’s asthma, it has been suggested that ATIs may activate innate immunity via the Toll-like receptor 4 pathway [28,29]. Furthermore, even when patients report the symptom onset or worsening after wheat ingestion, it is possible they are actually reacting to proteins in other foods ingested with wheat, high FODMAPs other than wheat, or experiencing a nocebo effect, in which symptoms caused by other factors are attributed to gluten. (“Pseudo NCGS” in Figure 2). In order to establish accurate diagnostic criteria for NCGS and identify “True NCGS,” a combination of several biomarkers including AGA IgG, antibodies to other wheat proteins, and inflammatory cytokines reflecting immune responses would be desirable. Furthermore, the elevation of AGA IgG in patients with suspected NCGS may be closely linked to increased intestinal permeability, often referred to as a “leaky gut.” Impairment of the intestinal barrier function allows various dietary proteins, including undigested gluten peptides, to penetrate the mucosal layer and encounter the submucosal immune system. While our study focused on AGA IgG, it is possible that these patients also exhibit sensitivities to other dietary antigens or wheat fructans (part of the FODMAPs) due to this shared underlying barrier dysfunction. Therefore, AGA IgG may serve as an indirect indicator of broader immune activation and intestinal permeability in a subset of NCGS patients.

Secondly, AGA IgG titers may fluctuate depending on dietary gluten intake. We have previously demonstrated that AGA IgG titers decrease following gluten restriction [30]. Thus, these titers may be influenced by the quantity of gluten ingested and the duration elapsed since ingestion. Although participants on a strict GFD were excluded from this study, it is possible that some participants who consumed only small amounts of gluten or less than healthy controls were included, due to the severity of their symptoms. These findings suggest that AGA IgG should also be assessed in combination with complementary markers, such as cytokines reflecting intestinal inflammation.

Previous reports have indicated that the seropositivity rate of AGA IgG in Western countries ranges from 2% to 8%. In our study, however, 13.3% of the 83 healthy controls were positive, suggesting a relatively higher prevalence in this population. Although dietary habits in Japan have become increasingly westernized, the annual per capita wheat consumption in Japan in 2023 was 31.0 kg, which remains substantially lower than that in Western countries, where it ranges from 100 to 200 kg. It is possible that the increasing intake of foods not traditionally consumed in large amounts may enhance immune responses to exogenous antigens and promote intestinal immune activation. If so, the prevalence of NCGS could potentially increase in the future.

Our results demonstrated that patients with suspected NCGS experience significantly higher psychological distress and lower QOL compared to healthy controls. Although these findings overlap with the clinical features of IBS, the objective identification of AGA IgG positivity in a subset of these patients suggests a specific immune-reactive background that may distinguish them within the broad spectrum of functional gastrointestinal disorders. While these differences are likely to reflect the chronic burden of symptoms and referral bias to our specialized clinic, rather than a direct causal link to gluten intake, they nonetheless reflect the clinical severity and substantial life disruption that lead these patients to seek specialized consultation. These findings suggest the existence of a subgroup of individuals in whom gluten-related concerns are associated with a variety of mental and physical symptoms, necessitating a multi-dimensional clinical approach.

Furthermore, the potential risks associated with a GFD must be considered. Gluten-free products tend to be lower in dietary fiber and essential micronutrients, such as folate and vitamins, as well as minerals including iron, zinc, magnesium, and calcium. Consequently, self-prescribed gluten restriction can easily lead to nutritional imbalances or deficiencies. To avoid such unnecessary dietary restrictions, it is essential to accurately identify “true NCGS” [16]. In the future, it is imperative to combine multiple biomarkers from various perspectives, extending beyond AGA IgG, to objectively characterize NCGS beyond subjective symptom reports.

## 5. Limitations

Several limitations of this study should be noted. First, celiac disease was excluded primarily based on anti-tTG IgA results. Although the prevalence of celiac disease is very low in Japan, the absence of data on total IgA and HLA-DQ2/DQ8 status means that rare cases of seronegative or IgA-deficient celiac disease might not have been fully captured. Second, clinical assessments relied on self-administered questionnaires, which represent subjective patient evaluations rather than objective physiological measurements. Third, this study could not definitively identify the specific causative agents for the reported symptoms. It remains unclear whether the reactions were triggered by gliadin, other wheat proteins such as ATIs and glutenin, FODMAPs, or a nocebo effect. Fourth, gluten exposure was not standardized prior to blood collection. Although participants on strict GFDs were excluded, some may have reduced their gluten intake due to symptom severity. Since AGA IgG titers fluctuate with gluten consumption, this lack of standardized intake might have influenced the serological results, potentially contributing to the low sensitivity observed in this study. Fifth, using healthy individuals as the sole control group limits our ability to evaluate the diagnostic specificity of AGA IgG against other symptomatic conditions like IBS.

## 6. Conclusions

In conclusion, our study demonstrates that AGA IgG has limited utility as a standalone biomarker for identifying patients with suspected NCGS. Furthermore, our findings indicate that AGA IgG levels do not directly correlate with the severity of clinical symptoms, psychological distress, or impairment of quality of life. It should be noted that elevated AGA IgG levels may not only represent a specific immune response to gluten but could also reflect underlying pathophysiology, such as increased intestinal permeability to various dietary antigens. Our findings demonstrate NCGS is a complex, heterogeneous condition characterized by substantial somatic and psychiatric burdens that extend beyond simple immune reactivity to gliadin. Given the modest sensitivity of AGA IgG, a multi-dimensional diagnostic approach incorporating clinical history, assessment of intestinal permeability markers, and a combination of immunological biomarkers is essential for accurately identifying “true NCGS”. Such precision is crucial not only for validating patient experiences but also for preventing unnecessary nutritional risks associated with self-prescribed GFDs.

## Figures and Tables

**Table 1:** Demographic and clinical characteristics of participants (suspected NCGS patients and healthy controls).

	Suspected NCGS	Controls	*p*-Value
	(*n* = 45)	(*n* = 83)	
	Medians (IQRs) or *n* (%)		
Female	29 (64.4)	42 (50.6)	1.4 × 10^−1^
Age, years	43.0 (28.5–51.8)	42.0 (36.0–51.0)	4.4 × 10^−1^
BMI, kg/m^2^	21.5 (17.6–25.4)	22.1 (20.2–24.2)	1.7 × 10^−1^
Allergies (non-wheat)	34 (75.6)	32 (38.6)	6.3 × 10^−5^ *
AGA IgG positive	15 (33.3)	11 (13.3)	7.0 × 10^−3^ *
QOL and Symptoms			
SF-36, Physical component summary	39.6 (30.6–50.3)	54.3 (51.1–56.2)	2.0 × 10^−8^ *
SF-36, Mental health component summary	39.7 (32.8–50.1)	58.0 (51.9–61.3)	7.1 × 10^−9^ *
PHQ-15	12.5 (8.0–17.5)	2.5 (1.0–5.0)	7.7 × 10^−9^ *
GAD-7	9.0 (3.5–12.8)	0.5 (0.0–2.8)	8.8 × 10^−11^ *
PHQ-9	9.5 (3.0–14.5)	1.0 (0.0–3.0)	7.6 × 10^−9^ *
IBS-QOL-J Total score	77.2 (48.5–86.6)	100.0 (100.0–100.0)	2.1 × 10^−19^ *

NCGS, Non-celiac gluten sensitivity; BMI, body mass index; IQRs, interquartile ranges; AGA IgG, anti-gliadin IgG antibody; SF-36, 36-Item Short Form Health Survey questionnaire; PHQ-15, Patient Health Questionnaire-15; GAD-7, Generalized Anxiety Disorder-7; PHQ-9, Patient Health Questionnaire-9; IBS-QOL-J, Japanese version of the irritable bowel syndrome quality of life. Data are expressed as medians (IQRs) for continuous variables and as frequencies (percentages) for categorical variables. * *p* < 0.05 for demographic characteristics, and *p* < 0.0083 for primary clinical and QOL scales, indicating statistical significance after Bonferroni correction for multiple comparisons.

**Table 2:** Prevalence of somatic symptoms (PHQ-15) in suspected NCGS patients and healthy controls.

	Suspected NCGS	Controls	*p*-Value
	(*n* = 45)	(*n* = 83)	
Symptoms			
Stomach pain	14 (34.1)	4 (4.8)	1.3 × 10^−5^ *
Back pain	11 (26.8)	2 (2.4)	8.3 × 10^−5^ *
Arms, legs, joints pain	16 (39.0)	13 (15.7)	3.8 × 10^−3^
Menstrual problems (women only) (*n* = 68)	9/26 (34.6)	2/42 (4.8)	1.9 × 10^−3^ *
Pain or problems during sexual intercourse	2 (4.9)	0 (0.0)	1.1 × 10^−1^
Headaches	13 (31.7)	9 (10.8)	4.2 × 10^−3^
Chest pain	5 (12.2)	0 (0.0)	3.3 × 10^−3^ *
Dizziness	7 (17.1)	2 (2.4)	6.0 × 10^−3^
Fainting spells	1 (2.4)	0 (0.0)	3.3 × 10^−1^
Feeling your heart pound or race	6 (14.6)	1 (1.2)	5.3 × 10^−3^
Shortness of breath	7 (17.1)	0 (0.0)	3.0 × 10^−4^ *
Constipation, loose bowels or diarrhea	30 (73.2)	5 (6.0)	5.5 × 10^−15^ *
Nausea, gas, or indigestion	27 (65.9)	3 (3.6)	2.7 × 10^−14^ *
Feeling tired or having low energy	24 (58.5)	9 (10.8)	1.6 × 10^−8^ *
Trouble sleeping	24 (58.5)	6 (7.2)	3.5 × 10^−10^ *

PHQ-15, Patient Health Questionnaire-15; NCGS, Non-celiac gluten sensitivity. * *p* < 0.0033 indicates statistical significance after Bonferroni correction for multiple comparisons of 15 somatic symptoms.

**Table 3:** Demographic and clinical profiles of the study population stratified by suspected NCGS and AGA IgG status.

	Suspected NCGS		Controls		
	AGA IgG Positive	AGA IgG Negative	AGA IgG Positive	AGA IgG Negative	*p*-Value
	(*n* = 15)	(*n* = 30)	(*n* = 11)	(*n* = 72)	
	Medians (IQRs) or *n* (%)				
Female	9 (60.0)	17 (56.7)	6 (54.5)	36 (50.0)	4.7 × 10^−1^
Age, years	36.0 (25.0–51.0)	43.5 (29.8–52.0)	49.0 (36.0–54.0)	41.0 (35.0–49.8)	5.4 × 10^−1^
BMI, kg/m^2^	20.7 (15.9–24.5)	21.3 (18.3–25.6)	22.0 (19.2–26.5)	22.0 (20.2–24.1)	5.6 × 10^−1^
Allergies (non-wheat)	10 (66.7) (AR = 1.2)	24 (80.0) (AR = 3.6 *)	2 (18.2) (AR = −2.3)	30 (41.7) (AR = −2.5)	3.0 × 10^−4^ *
diarrhea	10 (66.7) (AR = 3.8 *)	21 (70.0) (AR = 6.0 *)	0 (0.0) (AR = −2.2)	5 (6.9) (AR = −6.2 *)	3.0 × 10^−13^ *
abdominal pain	11 (73.3) (AR = 4.7 *)	17 (56.7) (AR = 4.7 *)	1 (9.1) (AR = −1.2)	3 (4.2) (AR = −6.4 *)	3.2 × 10^−5^ *
Meets diagnostic criteria for IBS	12 (80.0)	19 (63.3)	excluded	excluded	3.2 × 10^−1^
	(*n* = 14)	(*n* = 27)	(*n* = 11)	(*n* = 72)	
QOL and Symptoms					
SF-36, Physical component summary	36.9 (24.5–53.7) ^†^	43.4 (31.5–52.1) ^†, ‡^	52.5 (51.2–55.4)	54.6 (50.6–56.5)	5.3 × 10^−7^ *
SF-36, Mental health component summary	45.6 (33.9–51.1) ^†, ‡^	40.3 (33.5–52.9) ^†, ‡^	58.8 (57.3–62.1)	57.1 (51.4–60.9)	1.2 × 10^−7^ *
PHQ-15	13.0 (10.0–16.0) ^†, ‡^	8.5 (4.8–15.8) ^†, ‡^	3.0 (2.0–4.0)	2.0 (1.0–5.8)	1.5 × 10^−7^ *
GAD-7	8.0 (4.5–12.0) ^†, ‡^	8.0 (2.0–13.0) ^†, ‡^	0.0 (0.0–2.0)	1.0 (0.0–3.0)	2.7 × 10^−9^ *
PHQ-9	9.0 (3.0–16.3) ^†, ‡^	9.0 (2.0–13.0) ^†, ‡^	0.0 (0.0–2.0)	1.0 (0.0–3.0)	8.1 × 10^−8^ *
IBS-QOL-J Total score	79.0 (57.4–86.2) ^†^	77.9 (44.1–91.9) ^†, ‡^	100.0 (98.5–100.0)	100.0 (100.0–100.0)	1.6 × 10^−17^ *

NCGS, Non-celiac gluten sensitivity; AGA IgG, anti-gliadin IgG antibody; IQRs, interquartile ranges; BMI, body mass index; IBS, Irritable bowel syndrome; AR, adjusted residual; SF-36, 36-Item Short Form Health Survey questionnaire; PHQ-15, Patient Health Questionnaire-15; GAD-7, Generalized Anxiety Disorder-7; PHQ-9, Patient Health Questionnaire-9; IBS-QOL-J, Japanese version of the irritable bowel syndrome quality of life. Data are expressed as medians (IQRs) for continuous variables and as frequencies (percentages) for categorical variables. *: Statistical significance after Bonferroni correction for multiple comparisons. Specifically, for continuous variables, *p* < 0.0083 was applied for primary clinical/QOL scales and *p* < 0.05 for demographic characteristics; for categorical variables, significance was defined as an absolute adjusted residual ∣Z∣ > 2.58 based on Haberman’s analysis. Pairwise comparisons were performed using Dunn’s post hoc test with Bonferroni correction. ^†^ *p* < 0.05 vs. Control AGA IgG-negative group ^‡^ *p* < 0.05 vs. Control AGA IgG-positive group.

**Table 4:** Multivariable logistic regression analysis of factors associated with suspected NCGS.

Variable	B	S.E.	Wald	*p*-Value	OR (95% CI)
AGA IgG positive	1.74	0.75	5.40	2.0 × 10^−2^ *	5.71 (1.32–24.78)
Age	0.001	0.02	0.001	9.7 × 10^−1^	1.00 (0.95–1.05)
Female	0.87	0.69	1.58	2.1 × 10^−1^	2.38 (0.62–9.26)
BMI	−0.04	0.09	0.18	6.7 × 10^−1^	0.96 (0.81–1.15)
Allergy status (yes)	1.91	0.67	8.19	4.0 × 10^−3^ *	6.77 (1.83–25.00)
GAD-7 score	0.35	0.14	6.09	1.4 × 10^−2^ *	1.41 (1.07–1.86)
PHQ-9 score	0.07	0.11	0.39	5.3 × 10^−1^	1.07 (0.86–1.34)

NCGS, Non-celiac gluten sensitivity; B, unstandardized regression coefficient; S.E., standard error; Wald, Wald chi-square statistic; OR, odds ratio; CI, confidence interval; AGA IgG, anti-gliadin IgG antibody; BMI, body mass index; GAD-7, Generalized Anxiety Disorder-7; PHQ-9, Patient Health Questionnaire-9. * *p* < 0.05 indicates statistical significance.

## Data Availability

The data presented in this study are available on request from the corresponding author due to ethical considerations.

## Abbreviations

AGA IgG, anti-gliadin IgG antibody; ATIs, amylase-trypsin inhibitors; CI, confidence interval; ELISA, enzyme-linked immunosorbent assay; FODMAPs, fermentable oligo-, di-, and monosaccharide and polyol foods; GAD-7, Generalized Anxiety Disorder-7; IBS, irritable bowel syndrome; IBS-QOL-J, Japanese version of the irritable bowel syndrome quality of life; NCGS, non-celiac gluten sensitivity; NPV, negative predictive value; PHQ-9, Patient Health Questionnaire-9; PHQ-15, Patient Health Questionnaire-15; PPV, positive predictive value; QOL, quality of life; SD, standard deviation; SF-36, 36-Item Short Form Health Survey.
